# Progress in State Medicine

**Published:** 1899-03-04

**Authors:** 


### Progress in State Medicine.

(*Continued from page* 367.)

**Disinfecting Power of Alcohol.**—Epstein[7] finds a 50 per cent. solution of alcohol to have the maximum antiseptic power, any departure above or below this standard resulting in diminished efficiency. The germicidal action of absolute alcohol is practically nil. Alcoholic solutions of antiseptic substances are weaker than the aqueous solutions, but to this rule there are certain exceptions, *e.g.*, corrosive sublimate, lysol, carbolic acid, and thymol have greater effect in 50 per cent. alcoholic solution than when dissolved in water. Minervini's[8] results are almost identical with the above. He finds that although certain bacteria are killed by alcohol, yet spores are unaffected. Fifty to seventy per cent. alcohol has the most marked bactericidal power, whilst the property is least marked in absolute alcohol. The value of alcoholic solutions of disinfectants varies inversely as the strength of the alcohol. Goenner, in a series of careful experiments, failed to disinfect the hands with alcohol. Cultures taken from the surface of the skin, or from beneath the nails, after cleansing with various strengths of alcohol, gave rise to cocci and various organisms.

**Soaps as Disinfectants.**—Rideal[9] regards an olein basis as that most suitable for an antiseptic soap. There should be a moderate excess of alkali and no free fat or oil, as the presence of the latter strongly militates against germicidal action—witness Koch's discovery that carbolised oil has no antiseptic value. Simple unmedicated curd soaps have an antiseptic value. In 1 per cent. solution many germs are killed in an hour, and in 2 per cent. solution B. coli dies in six hours. Cold solutions are more active than hot.

With regard to antiseptic soaps acids and free halogens are incompatible, the one being neutralised and the other combining with the fat. Boric acid is unsuitable, and the oleates do not mix well with soap. Oxides, fluorides, and sulphides give good results. Mercury forms an insoluble mercuric oleate, but the double iodide of potash and mercury forms an excellent antiseptic, being compatible with strong alkalis and not precipitating albumen. Carbolic soap generally contains about 10 per cent. of the acid, and, like coal tar soap and others containing antiseptic substances, has a feeble germicidal action, more pronounced on B. coli than on the cocci. Organisms require a prolonged exposure varying from one to four hours to be killed by 1 per cent. soap solution.

Reithoffer[10] refers to the different behaviour of various germs to soap solutions. Thus anthrax bacilli are easily killed in weak solutions, but typhoid and cholera germs are far more resistant. He notes that in Behring's opinion the disinfectant power of a soap is dependent on its alkalinity. Experimenting with common soft soap, with scented almond soap, and with a patent potash soap, Reithoffer found that they all had a high degree of disinfecting power against typhoid bacilli, B. coli, and the cholera organisms, but their action is very feeble against pus cocci. Staphylococcus pyogenes aureus will remain unchanged for an hour or more in 18 to 20 per cent. soap solution, whilst a 10 per cent. solution of the same soap will kill typhoid bacilli in one minute. Experiments with carbolic soaps and lysol soap showed that although the germicidal power as tested on cocci was slightly increased, yet it was much weaker than was a solution of the disinfectant without the soap. From these observations it may be concluded that in surgical practice the use of disinfectant soap is inefficient, the better plan being to wash first with soap and afterwards to apply an antiseptic lotion.

